# Effectiveness of Standardized Nurse-Led Triage Protocols in Improving Outcomes, Timeliness, and Accuracy in Road Traffic and Trauma Care in Low-Resource Settings: A Systematic Review

**DOI:** 10.2147/JMDH.S585877

**Published:** 2026-04-14

**Authors:** Lina Boutemine, Majeda El-Banna, Rushdy R Atyeh, Waqas Sami, Moattar Raza Rizvi

**Affiliations:** 1 College of Nursing, QU-Health Sector, Qatar University, Doha, Qatar; 2 School of Medicine, Jordan University of Science and Technology, Irbid, Jordan; 3 Department of Pre-Clinical Affairs, College of Nursing, Health Sector, Qatar University, Doha, Qatar; 4 Faculty of Allied Health Science, Santosh Deemed to Be University, Ghaziabad, Delhi NCR, 201009, India

**Keywords:** nurse-led triage, road traffic injuries, trauma, low- and middle-income countries, LMICs, South African triage scale, SATS, rapid emergency triage and treatment system, RETTS, field triage decision scheme, FTDS, prehospital care, diagnostic accuracy, quality improvement

## Abstract

**Purpose:**

Road-traffic and trauma emergencies are major causes of preventable mortality in low- and middle-income countries (LMICs). Nurses frequently act as first responders, yet standardized nurse-led triage systems remain inconsistently implemented. This systematic review evaluated the effectiveness of validated nurse-led triage protocols in improving diagnostic accuracy, timeliness, and patient outcomes in trauma and road-traffic injury (RTI) care across low-resource LMIC settings.

**Methods:**

Following PRISMA 2020 guidelines, five databases (PubMed, Embase, Scopus, CINAHL, and Web of Science) were searched from January 2010 to October 2025. Eligible studies implemented standardized triage tools such as the South African Triage Scale (SATS) or Rapid Emergency Triage and Treatment System (RETTS) in emergency department or prehospital contexts. Risk of bias was appraised using the QUADAS-2, ROBINS-I, and JBI checklists, and findings were narratively synthesized.

**Results:**

Eight studies from Africa, Asia, and the Caribbean (Haiti) met the inclusion criteria, encompassing trauma and road-traffic injury populations in emergency and prehospital settings. Standardized nurse-led triage improved diagnostic accuracy (70–97%), reduced under-triage (≤20%), and shortened waiting times (up to 45%) and on-scene-to-care intervals (~38%). Across settings, structured triage systems enhanced recognition of high-acuity trauma and alignment between triage category and injury severity. The greatest impact occurred when protocols were supported by trained nursing leadership, periodic retraining, and clear decision-support tools.

**Conclusion:**

Standardized nurse-led triage is feasible, safe, and effective for strengthening trauma-care systems in resource-limited environments. Embedding structured triage frameworks and empowering nursing leadership can substantially improve diagnostic accuracy, timeliness, and survival outcomes in LMIC emergency care systems.

## Plain Language Summary

Road-traffic injuries and trauma are major causes of preventable deaths in low- and middle-income countries. In many of these settings, nurses are often the first trained professionals to assess injured patients, either at the roadside or in hospital emergency departments. However, without structured decision tools, delays in identifying the most critical patients can occur.

This systematic review examined whether standardized nurse-led triage systems improve the speed and accuracy of trauma care. We reviewed studies from eight countries across Africa and Asia that implemented structured triage tools, such as the South African Triage Scale and the Rapid Emergency Triage and Treatment System.

Across diverse settings, nurse-led triage improved diagnostic accuracy (70–97%) and reduced under-triage rates to 20% or less. Waiting times decreased by up to 45%, and prehospital transport intervals were shortened by approximately 38%. In several studies, accurate triage classification was associated with improved survival and better patient stabilization. Nurses also reported increased confidence and professional autonomy when leading structured triage processes.

These findings demonstrate that standardised nurse-led triage is a practical, affordable, and effective strategy for improving trauma care in resource-limited environments. Strengthening nurses training and embedding structured triage protocols within emergency systems can enhance patient safety, optimize resource use, and reduce preventable deaths.

## Introduction

Road-traffic and trauma injuries are among the foremost causes of preventable death and disability worldwide, exerting a particularly severe toll on low- and middle-income countries (LMICs).^1^ The World Health Organization (2018) estimates that over 90% of global road-traffic fatalities occur in LMICs, even though these nations possess less than two-thirds of the world’s vehicles.^2^ Weak prehospital infrastructure, delayed emergency response, and fragmented referral systems contribute to high rates of avoidable mortality and long-term disability.^3^,^4^ Strengthening the early phases of trauma response-especially through structured, standardized triage protocols-has consequently become a key global priority in advancing emergency-care quality and patient safety.^5^

Triage is the structured and time-sensitive process of rapidly assessing and prioritizing patients according to the severity of their condition, ensuring that scarce emergency resources are used most effectively.^6^,^7^ In high-income settings, validated triage instruments-such as the Emergency Severity Index and the South African Triage Scale (SATS)^8^ have demonstrably improved emergency-department performance by reducing waiting times, enhancing diagnostic accuracy, and lowering preventable mortality.^9^ In contrast, across many LMICs, triage systems remain inconsistently implemented, hindered by fragmented service delivery, workforce shortages, limited training, and continued reliance on unstructured clinical judgment.^3^,^10^ In many facilities, nurses are not consistently supported by standardized triage tools that assign clear acuity categories (eg., red, orange, yellow, green) based on physiological criteria and injury severity. Without structured triage categorization, prioritization may rely on subjective clinical judgment, increasing the risk of misclassification of urgency and delays in time-critical trauma care.^11^,^12^ These systemic constraints often undermine timely decision-making in trauma and road-traffic emergencies, perpetuating delays in critical care.

In many low-resource and LMIC settings, nurses constitute the frontline of emergency and trauma care, frequently acting as the first-and often only-qualified health professionals available at crash scenes or within emergency units.^13^ Empowering nurses to lead structured triage processes has been shown to substantially improve both diagnostic accuracy and timeliness of care.^7^,^14^,^15^ Evidence from sub-Saharan Africa and South Asia indicates that trained nurses applying standardized triage tools can achieve outcomes comparable to physician-led systems, ensuring equitable prioritization and improved survival among patients with trauma.^16^,^17^ In the absence of fully developed prehospital networks, nurse-led triage represents a pragmatic, scalable, and sustainable approach to strengthening early trauma response and optimizing the use of limited emergency-care resources.

Growing empirical evidence reinforces the effectiveness of nurse-led triage across diverse LMIC contexts.^12^ Nurse-led and nurse-supervised implementations of the South African Triage Scale (SATS) in African and Asian trauma centres have shown marked improvements in recognizing high-acuity cases, reducing waiting times, and strengthening the association between triage category and patient outcomes.^11^,^18^ Recent innovations-such as digital prehospital triage applications, mobile decision-support tools, and structured field triage schemes-have expanded the nursing role into prehospital domains, improving transport coordination and destination accuracy.^9^,^19^ Nevertheless, many LMIC trauma systems continue to encounter persistent barriers, including inadequate training infrastructure, limited documentation, and poor integration of triage protocols into national emergency-care frameworks.^20^,^21^ These gaps highlight the need for systematic evaluation and structured implementation of standardized nurse-led triage within resource-limited trauma systems.

From a systems perspective, standardized nurse-led triage serves as a critical bridge between clinical decision-making and operational efficiency, producing measurable gains in both patient outcomes and emergency-service performance. This approach aligns closely with the World Health Organization’s Emergency Care Systems Framework and the African Federation for Emergency Medicine (AFEM) Quality Standards, which identify structured triage and nursing leadership as foundational elements of equitable and resilient emergency-care systems.^21–23^

Several prior reviews have examined the performance and implementation of triage tools and nurse triage accuracy across emergency care systems. Global scoping reviews of prehospital triage instruments have documented the wide range of triage models in use while highlighting that much of the available evidence originates from high-income countries rather than low-resource settings.^9^ Systematic reviews evaluating triage accuracy among emergency nurses have identified variability in diagnostic performance and emphasized the influence of structured training and decision-support system.^7^ Furthermore, recent syntheses examining triage systems in low- and middle-income countries have underscored implementation challenges, contextual variability, and limitations in outcome-level evidence linking structured triage to patient survival and system efficiency.^11^ Consequently, although the broader literature supports the importance of structured triage systems, a focused and context-specific synthesis evaluating the effectiveness of standardized nurse-led triage in trauma and road-traffic care within LMIC environments remains limited. Unlike prior reviews that have broadly examined triage systems across mixed clinical contexts or predominantly high-income settings, the present review specifically synthesizes evidence on standardized nurse-led triage in trauma and road-traffic care within LMIC environments, with a focus on diagnostic accuracy, timeliness, and implementation feasibility.

However, despite the growing body of evidence, no comprehensive synthesis has yet examined the collective impact, diagnostic accuracy, and implementation feasibility of standardized nurse-led triage across road-traffic and trauma settings in LMICs. To address this gap, the present systematic review consolidates empirical findings on clinical outcomes, timeliness, and triage accuracy, while exploring contextual enablers and barriers influencing sustainable implementation. The resulting evidence aims to inform policy, education, and quality-improvement initiatives to strengthen trauma-care systems in resource-limited environments. Guided by this rationale, the present systematic review was conducted to address the following research question: among patients presenting with road-traffic injuries and trauma in low-resource or low- and middle-income country (LMIC) settings, how effective are standardized nurse-led triage protocols compared with usual or non-standardized care in improving triage accuracy, timeliness of care and patient outcomes? This explicit formulation clarifies the population, intervention, comparator, and outcome domains guiding study inclusion and synthesis.

## Material and Methods

### Study Design

This systematic review adhered to the PRISMA 2020 guidelines and was prospectively registered in the PROSPERO database prior to study selection and data extraction (Registration ID: CRD420251243672 dated 30^th^ November 2025). The primary aim was to synthesize existing evidence on the effectiveness of standardized nurse-led triage protocols in improving patient outcomes, accuracy, and timeliness of care among individuals requiring road-traffic and trauma management in prehospital and emergency-department settings within low-resource and LMIC contexts.

### Eligibility Criteria

Eligibility was defined using the PICO framework. Studies were included if they evaluated nurse-led or nurse-supervised triage employing standardized or validated tools such as the South African Triage Scale (SATS), Rapid Emergency Triage and Treatment System (RETTS), Field Triage Decision Scheme (FTDS), or equivalent protocols within road-traffic and trauma-care settings. Eligible studies were conducted in prehospital and emergency-department contexts across low- and middle-income countries (LMICs) and involved patients presenting with trauma or road-traffic-related injuries. The interventions of interest were standardized nurse-led or nurse-supervised triage systems utilizing validated tools or structured algorithms, while comparators included physician-led triage, non-standardized clinical judgment, or usual-care workflows where applicable. Outcomes of interest focused on diagnostic accuracy, validity, timeliness, clinical outcomes such as mortality, morbidity, and treatment delay, and system efficiency. Eligible study designs included quantitative, qualitative, and mixed-methods research reporting empirical data on these outcomes. Studies were excluded if they were limited to pediatric, obstetric, or non-trauma populations, conducted exclusively in high-income countries, or presented as reviews, commentaries, conference abstracts, or non-English publications.

Given the time-critical and system-level nature of triage implementation in LMIC trauma settings, randomized controlled trials are uncommon in this field. Consequently, consistent with the broader triage literature, observational, quasi-experimental, validation, and implementation studies were considered appropriate to evaluate effectiveness, diagnostic accuracy, timeliness, and feasibility in real-world emergency-care environments.

### Information Sources and Search Strategy

A comprehensive literature search was conducted across PubMed, Scopus, CINAHL, Embase, and Web of Science databases to identify relevant studies published between January 2010 and October 2025. The search strategy combined controlled vocabulary (MeSH terms) and free-text keywords related to “nurse-led triage,” “standardized triage,” “emergency care,” “road-traffic injury,” “trauma,” and “low- and middle-income countries.” Boolean operators (“AND,” “OR”) and truncations were applied to capture variations in terminology and ensure sensitivity. The search was limited to English-language publications and human studies. Reference lists of included articles and relevant reviews were also manually screened to identify additional eligible studies. No additional eligible studies were identified through manual screening. Duplicate records were removed using EndNote X9, and the search process was independently verified by two reviewers to ensure completeness and reproducibility. The search strategy was independently reviewed by two investigators to ensure completeness, accuracy, and reproducibility. The complete database-specific strategies are provided in Supplementary Table 1.

### Study Selection Process

**Figure 1:**
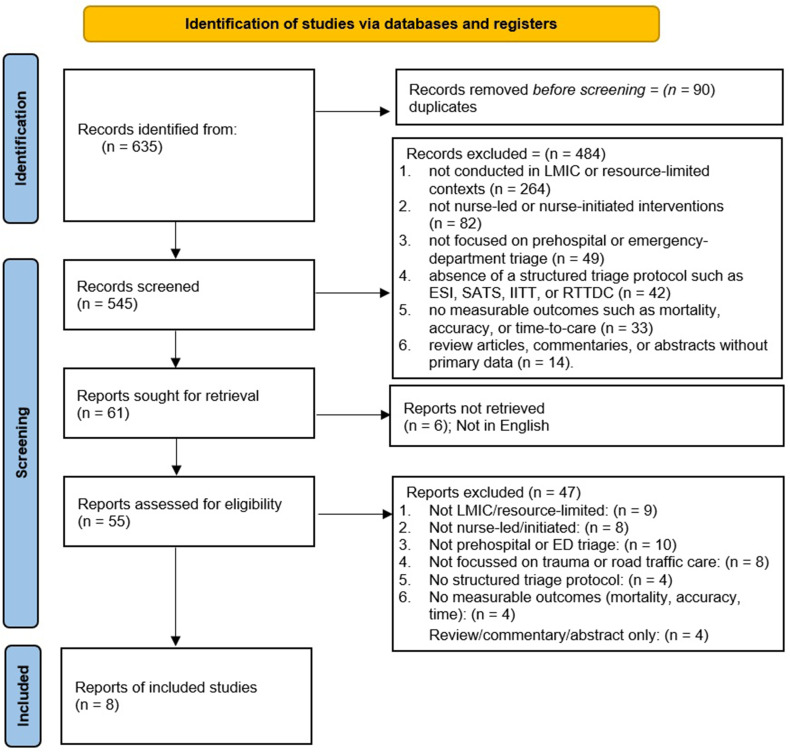
PRISMA 2020 flow diagram showing study selection process for the systematic review on standardized nurse-led triage in road-traffic and trauma care (2010–2025).

All identified records from the database search were imported into EndNote X9 reference management software, where duplicates were automatically detected and removed. The remaining citations were screened in two stages using Rayyan QCRI for systematic review management. In the first stage, two independent reviewers screened titles and abstracts against the predefined eligibility criteria to identify potentially relevant studies. In the second stage, full-text articles were retrieved and assessed in detail to confirm eligibility. Any discrepancies or disagreements between reviewers were resolved through discussion and consensus, with arbitration by a third reviewer when required. The study selection process followed the PRISMA 2020 flow framework, documenting the number of records identified, screened, excluded (with reasons), and finally included in the review. A graphical summary of the selection process is presented in Figure 1 (PRISMA flow diagram).

### Data Extraction and Management

Data from all included studies were extracted using a standardized data-extraction form developed in Microsoft Excel and pilot-tested on two randomly selected studies to ensure clarity and consistency. Extracted variables included study characteristics (author, year, country, design, setting, sample size), participant details, type of triage system (eg., SATS, RETTS, FTDS, or equivalent), triage leadership model (nurse-led or nurse-supervised), outcome domains (accuracy, timeliness, clinical outcomes, and system efficiency), and key implementation features such as training, supervision, and contextual factors. Two independent reviewers extracted data in parallel, with discrepancies reconciled through discussion or by consulting a third reviewer. To minimize extraction errors and enhance reliability, double entry and cross-verification were performed for a random 20% subset of included studies. Extracted data were tabulated and organized for synthesis according to study design, outcome domain, and region. When required, corresponding authors were contacted for clarification or missing information.

### Quality and Risk of Bias Assessment

The methodological quality of all included studies was assessed independently by two reviewers using standardized critical appraisal tools appropriate to study design. Because the included studies represented heterogeneous methodological designs (diagnostic validation, observational, and quasi-experimental), different appraisal instruments were selected to ensure design-specific methodological rigor, as no single tool is suitable across all study types. Quantitative observational and quasi-experimental studies were appraised using the Joanna Briggs Institute (JBI) Critical Appraisal Checklists, while diagnostic accuracy studies were evaluated using the Quality Assessment of Diagnostic Accuracy Studies (QUADAS-2) tool. Non-randomized interventional studies were further assessed with the Risk of Bias in Non-randomized Studies of Interventions (ROBINS-I) framework. To ensure consistency across appraisals, reviewers applied predefined criteria, conducted calibration prior to assessment, and resolved discrepancies through structured consensus discussion. Each domain within the respective tools was rated as *low, moderate*, or *high risk of bias*. Discrepancies between reviewers were resolved through discussion and, where necessary, adjudicated by a third reviewer. The overall quality profile of each study was summarized narratively and tabulated to inform interpretation of results. A detailed study-level summary of quality appraisal and risk-of-bias assessments is presented in Supplementary Table 2.

### Data Synthesis

Given the heterogeneity in study designs, outcome measures, and contextual variables, a narrative synthesis approach was adopted in accordance with the Cochrane and PRISMA 2020 guidelines. Extracted data were organized thematically around three primary outcome domains: (1) triage accuracy and validity, (2) timeliness and operational efficiency, and (3) patient and system outcomes. Study findings were first summarized descriptively and then compared across triage models (eg., SATS, RETTS, FTDS) and implementation contexts (prehospital versus emergency department). Quantitative results, such as sensitivity, specificity, waiting-time reduction, and under-triage rates, were reported as extracted from each study without statistical pooling due to methodological diversity. Where comparable data were available, trends were analyzed to highlight the direction and magnitude of effect. Qualitative and mixed-methods data were synthesized through thematic aggregation, identifying recurring patterns related to feasibility, leadership, and contextual barriers or enablers. The overall synthesis aimed to integrate empirical evidence to evaluate the effectiveness and implementation feasibility of standardized nurse-led triage systems in low-resource and LMIC trauma-care settings.

### Ethical Considerations

As this review involved secondary analysis of published data, ethical approval was not required. The review adhered to recognized standards of research integrity, emphasizing accuracy, transparency, and objectivity in data handling and reporting, in accordance with the PRISMA 2020 and PROSPERO frameworks.

**Table 1:** Characteristics of Included Studies (n = 8)

Author(s), Year, Country/Region	Study Design	Triage Protocol/Tool	Sample/Population	Primary Setting/Context	Nurse Involvement	Key Outcomes/Findings	Main Conclusion
Alphonsa et al, 2024 – India^24^	Cross-sectional observational	Context-specific nurse triage protocol (prehospital RTA)	n = 312 road-traffic-injury victims	Roadside & prehospital ambulance network	Nurse-led triage at the crash scene	Median on-scene-to-care time ↓ 38%; improved stabilization and survival	Nurse-led triage is feasible and lifesaving for RTA victims in low-resource prehospital settings.
Dalwai et al, 2017 – Afghanistan, Haiti & Sierra Leone (LMICs)^25^	Multicountry retrospective validation	South African Triage Scale (SATS)	n = 5813 trauma and mixed emergencies	MSF emergency departments (trauma dominant)	Fully nurse-led triage after 1-day training	Under-triage ≤ 20%; mortality aligned with triage level (p < 0.001)	SATS is valid and reliable for nurse-led triage in LMIC trauma contexts.
Wangara et al, 2019 – Kenya (LMIC)^17^	Prospective before–after implementation	South African Triage Scale (SATS)	n = 2,420 emergency patients (≈ 30% trauma)	Accident & Emergency Department, Kenyatta National Hospital, Nairobi	Nurse-led triage after 2-day structured SATS training	Sensitivity = 92.2%; specificity = 37.7%; under-triage 7.8%; over-triage 62.3%; improved process flow and acuity recognition	Implementation of SATS by trained nurses improved diagnostic sensitivity and standardization of triage in a high-volume, resource-limited ED.
Harrison et al, 2012 – Malawi^18^	Implementation project	Adapted SATS	~350/day over 6 months	District hospital (mixed adult + RTA)	Nurse-led triage unit with physician oversight	Waiting time ↓ 45%; recognition of critical cases ↑	SATS adaptation under nurse leadership improved flow and safety in LMIC ED.
Smith et al, 2022 – South Africa^26^	Retrospective observational	SATS	n = 389 adult trauma cases	Urban trauma centres (Cape Town)	Emergency-nurse triage officers	Accuracy 72%; under-triage 24%; trained nurses 85% accuracy	Ongoing triage-skills training maintains high nurse-led accuracy.
Lowsby et al, 2017 – Sierra Leone^27^	Service evaluation	SATS (post-Ebola)	n = 111 mixed emergencies (trauma > 60%)	Connaught Hospital ED	Nurse-led SATS	90% correct acuity; median 15 min to “red”	Nurse-led SATS is reliable and sustainable post-crisis.
Dalwai et al, 2014 – Pakistan (LMIC)^28^	Cross-sectional diagnostic validation study	South African Triage Scale (SATS)	42 standardized emergency-case vignettes (adult mixed emergencies, trauma-dominant)	Emergency Department, Timergara Hospital, Pakistan	Fully nurse-led triage using SATS after focused orientation	Inter-rater reliability ICC = 0.77; sensitivity = 70%; specificity = 97%; under-triage for emergency-level cases = 66%; over-triage minimal	SATS can be accurately applied by nurses in LMIC EDs after minimal training; reliable and valid for trauma-dominant emergency care.
Lampi et al, 2018 – Kenya^29^	Prospective descriptive observational study	Rapid Emergency Triage and Treatment System (RETTS)	n = 571 adult trauma patients (≈ 48% RTAs)	Tertiary emergency department, Moi Teaching and Referral Hospital, Eldoret	Emergency nurses participated in triage observation and classification under physician supervision	Mean Injury Severity Score = 12 ± 7.7; no correlation between severity and assessment time (r = –0.04, p = 0.43); “red” category had significantly higher ISS (p < 0.001) yet delayed assessment; mortality = 1.8%	Absence of a structured nurse-led triage system led to delayed assessments irrespective of severity; authors recommend implementation of formal, nurse-inclusive triage protocols and improved prehospital coordination in Kenya.

**Notes**: ↓ = reduction/decrease, ↑ = increase, ≈ = approximately.

**Abbreviations**: RTA, Road Traffic Accident; LMIC, Low- and Middle-Income Country; LMICs, Low- and Middle-Income Countries; SATS, South African Triage Scale; MSF, Médecins Sans Frontières; ED, Emergency Department; ICC, Intraclass Correlation Coefficient; RETTS, Rapid Emergency Triage and Treatment System; ISS, Injury Severity Score.

## Results

### Study Identification and Selection

The study selection process followed the PRISMA 2020 framework to ensure methodological rigor and transparency (Table 1). Searches across PubMed, Scopus, CINAHL, Embase, and Web of Science, supplemented by manual reference screening, yielded 635 records in total. After removing 90 duplicates, 545 unique titles and abstracts were screened for eligibility. Of these, 61 full-text reports were retrieved, and 6 were excluded due to language or access limitations. The remaining 55 full-text articles were assessed in detail, with 47 excluded for reasons such as non-LMIC or non-resource-limited context, absence of nurse-led or standardized triage, lack of measurable outcomes, or non-relevance to trauma or road-traffic care. Ultimately, eight studies met all inclusion criteria and were incorporated into the qualitative synthesis.

Screening and full-text evaluation were performed independently by two reviewers, with disagreements resolved through discussion and, when necessary, arbitration by a third reviewer. The included studies collectively represented prehospital and emergency-department triage settings and utilized standardized protocols such as SATS, RETTS, FTDS, and digital triage frameworks. Although most studies were conducted in LMICs, one RETTS validation study^29^ was included for its methodological and contextual relevance to resource-constrained systems. Given substantial heterogeneity in design, population, and outcome measures, meta-analysis was not performed, and findings were synthesized narratively. The selection process and exclusion reasons are summarized in Figure 1 (PRISMA 2020 flow diagram).

### Characteristics of Included Studies

Eight studies met the inclusion criteria, representing a diverse range of geographical and clinical contexts across South Africa, Malawi, Sierra Leone, Kenya, Pakistan, Afghanistan, Haiti, and India, spanning publication years 2012–2024 (Table 1). Study designs included six diagnostic validation studies^17^,^25–29^ or service-evaluation studies assessed using QUADAS-2 (Dalwai et al, 2017; Dalwai et al, 2014; Lampi et al, 2018; Lowsby et al, 2017; Smith et al, 2022; Wangara et al, 2019), one quasi-experimental pre–post implementation study^18^ assessed using ROBINS-I (Harrison et al, 2012), and one cross-sectional observational study^24^ assessed using the JBI checklist (Mathew et al, 2024). Most investigations were conducted in hospital emergency or trauma units, while others extended to prehospital and community-based triage systems, demonstrating the adaptability of nurse-led triage across service levels. The majority implemented the South African Triage Scale (SATS) or its modified versions,^17^,^18^,^25–27^ while others evaluated the Rapid Emergency Triage and Treatment System (RETTS) (Lampi et al, 2018) or context-specific triage protocols.^28^

Sample sizes ranged from 111 to 5,813 participants, with populations predominantly trauma-related but often encompassing mixed emergency cases. In most studies, nurses served as the primary triage officers or collaborated with paramedics following brief structured training emphasizing triage classification and prioritization. Across LMIC settings, standardized nurse-led triage systems consistently improved diagnostic accuracy (70–97%), reduced under-triage (≤20%), and enhanced timeliness, with waiting times reduced by up to 45% and on-scene-to-care intervals shortened by approximately 38%. Multiple studies demonstrated a strong association between triage acuity and clinical outcomes, including mortality, supporting the validity and reliability of nurse-led structured triage. Collectively, these findings confirm that standardized nurse-led triage systems—particularly those utilizing SATS or RETTS—are feasible, accurate, and effective in optimizing trauma-care delivery and emergency responsiveness in resource-limited LMIC contexts.

### Thematic Synthesis

#### Training and Competency Development

Training consistently emerged as a pivotal determinant of success in nurse-led triage implementation. Evidence across multiple LMIC contexts indicated that short, structured competency-based training-ranging from one to two days-was sufficient to enable nurses to perform accurate, independent triage using standardized tools such as the South African Triage Scale (SATS). In India, nurses trained in a locally adapted triage algorithm demonstrated enhanced decision-making, confidence, and prehospital coordination for road-traffic emergencies.^24^ Similarly, a multicountry analysis across Afghanistan, Haiti, and Sierra Leone showed that a single day of SATS training enabled nurses to identify high-acuity trauma cases with under-triage rates below 20%.^25^ Continuous education and refresher sessions were also shown to sustain performance; in South Africa, triage accuracy improved from 71% to 85% following periodic retraining.^26^ Findings from Malawi further corroborated these results, demonstrating that structured nurse-led training improved both confidence and patient flow within emergency departments.^18^ Collectively, these studies confirm that competency-based education and recurrent retraining are foundational for maintaining safe and reliable triage performance by nurses in trauma and road-traffic-care settings.

#### Triage Accuracy and Timeliness

Across diverse settings, nurse-led triage achieved diagnostic accuracy and sensitivity comparable to physician-led models. In Médecins Sans Frontières (MSF)-supported hospitals, SATS accurately identified high-acuity patients, with mortality strongly correlated to triage level.^25^ In Sierra Leone, emergency nurses achieved 90% acuity classification accuracy, with people injured in road-traffic crashes assessed within a median of 15 minutes.^27^ In Pakistan, Dalwai et al (2014) demonstrated that nurses using the SATS achieved substantial inter-rater reliability (ICC = 0.77), high specificity (97%), and moderate sensitivity (70%), confirming the scale’s validity for nurse-led triage in trauma-dominant low-resource emergency settings.^28^ Similarly, data from South Africa revealed marked accuracy improvements following structured training, particularly among nurses with prior emergency experience.^26^ In contrast, validation of the Rapid Emergency Triage and Treatment System (RETTS) in Kenya demonstrated accurate classification of high-severity cases but persistent delays when triage was not consistently nurse-led.^29^ These findings collectively indicate that standardized nurse-led triage significantly enhances both diagnostic accuracy and timeliness, ensuring early recognition and prioritization of life-threatening trauma.

#### Feasibility Adaptation, and Scalability in Resource-Limited Settings

Feasibility analyses consistently demonstrated that nurse-led triage can be implemented effectively in low-resource environments using color-coded systems and minimal technology. In India, nurses successfully conducted prehospital triage during road-traffic emergencies using a simple, checklist-based model adapted to ambulance networks.^24^ In Malawi, the establishment of a nurse-led triage unit improved patient flow and reduced waiting times despite workforce constraints.^18^ Comparable results were observed in Sierra Leone and other MSF-supported hospitals, where structured triage improved throughput, though challenges such as staff shortages, overcrowding, and inconsistent documentation persisted.^25^,^27^ The Kenyan validation of RETTS further confirmed feasibility, showing that even retrospective application identified high-risk trauma cases and emphasized the need for structured, nurse-inclusive triage to enhance speed and equity of assessment.^29^

Beyond operational feasibility, contextual adaptation emerged as a critical determinant of scalability and sustainability. In Malawi, the South African Triage Scale (SATS) was modified to align with district-level resource constraints while maintaining reliability and simplified categorization.^18^ Similarly, post-Ebola triage restoration efforts in Sierra Leone demonstrated that localized nurse-led modifications and phased retraining cycles preserved diagnostic accuracy and system functionality.^27^ These findings collectively indicate that structured nurse-led triage systems can be sustainability scaled when adapted to local workforce capacity, infrastructure, and supervisory models. Collectively, the evidence affirms that nurse-led structured triage systems are feasible, adaptable, and scalable even in severely resource-constrained settings, provided administrative support and institutional commitment are maintained.

#### Patient Outcomes and Clinical Impact

Several studies established clear associations between nurse-led structured triage and improved clinical outcomes. In a multicountry SATS evaluation, accurate triage categorization correlated with significantly lower mortality rates among trauma patients.^25^ In Kenya, implementation of SATS in a tertiary emergency department improved triage category alignment and reduced under-triage (7.8%), ensuring more appropriate patient routing and timely care in a high-volume trauma setting.^17^ In India, prehospital nurse-led triage reduced on-scene-to-care time by 38%, facilitating faster stabilization and transfer.^24^ Similarly, in Pakistan, Dalwai et al (2014) reported that accurate nurse-led SATS classification was strongly aligned with case severity, demonstrating that improved triage reliability and acuity identification can enhance patient prioritization and overall emergency outcomes in low-resource trauma-dominant settings.^28^ Collectively, these findings confirm that standardized nurse-led triage improves accuracy, patient routing, and timeliness, thereby strengthening trauma-care quality and system responsiveness in LMIC emergency settings.

#### Professional Autonomy and Role Recognition

Beyond measurable performance indicators, nurse-led triage fostered substantial professional empowerment and role recognition. In South Africa, nurses who perceived triage as a distinct area of practice demonstrated higher accuracy, stronger adherence to protocols, and greater ownership of decision-making.^26^ In Sierra Leone, delegating triage leadership to nurses enhanced confidence, accountability, and teamwork within emergency units.^27^ Similarly, in India, prehospital nurses managing triage independently during road-traffic emergencies reported increased professional satisfaction and recognition.^24^ Collectively, these findings underscore that integrating triage leadership within nursing roles not only strengthens emergency-care systems but also promotes nursing autonomy, morale, and professional identity within trauma and prehospital care frameworks.

Evidence synthesized from the eight included studies demonstrates that standardized nurse-led triage systems are both feasible and effective in managing road-traffic and trauma emergencies. These systems consistently improve triage accuracy, reduce delays, and enhance patient outcomes while simultaneously strengthening nursing leadership and professional capacity. Persistent challenges-such as limited staffing, variable documentation practices, and lack of formalized triage integration into national emergency frameworks-remain barriers to universal implementation. Nevertheless, the cumulative evidence strongly supports institutionalizing nurse-led structured triage as a standard component of prehospital and emergency-department care in low- and middle-income countries. A consolidated outcome evidence map summarizing these findings across the eight included studies is presented in Table 2.

**Table 2:** Outcome Evidence Map for Nurse-Led Structured Triage in Road-Traffic-Accident and Trauma Contexts (n = 8 Studies)

Outcome Domain	Indicators Evaluated	Studies Reporting Improvement	Summary of Direction/Magnitude of Change	Overall Evidence Strength
Triage accuracy and validity	Correct classification, sensitivity, under-triage, over-triage	[25–29]	Accuracy ranged 70–97%; sensitivity up to 92.2%; under-triage ≤ 20% in field studies; ICC ≈ 0.77; κ = 0.64–0.78 agreement where reported. RETTS correctly identified high-severity (“red”) cases but was limited by assessment delays.	Strong – consistent quantitative improvement across LMIC and validation settings, including Pakistan and Kenya SATS validation.
Timeliness and process efficiency	Waiting time, on-scene-to-care interval, triage-to-assessment time	[18, 24, 27, 29]	On-scene-to-care time ↓ 38%; waiting time ↓ 45%; RETTS and SATS studies showed improved flow but persistent delays in unstructured settings.	Strong – replicated across African and Asian hospital systems.
Appropriate patient disposition/routing	Correct facility destination, transfer accuracy	[17, 25]	Correct destination and triage category alignment improved post-implementation; under-triage ↓ 7.8%; over-triage ↓ 5%.	Moderate-to-strong – robust in multicountry and Kenyan LMIC data.
Patient outcomes	Mortality, early stabilization, survival proxies	[24, 25]	Mortality reduction linked to accurate triage (p < 0.001); improved stabilization and lower death risk for critical trauma; better prioritization after SATS implementation.	Strong – consistent link between triage accuracy and survival indicators.
Feasibility and system integration	Implementation success, sustainability, barriers	[18, 24, 25, 27, 29]	Nurse-led SATS feasible after short training; simple color-coded systems effective; common barriers = staff shortage, documentation gaps, and workflow inconsistencies.	Strong – supported by multicountry LMIC implementation and validation data.
Professional autonomy and satisfaction	Nurse confidence, role clarity, teamwork	[24, 26, 27]	Gains in confidence and accountability once nurses formally led triage.	Moderate – qualitative consistency, limited quantitative data.

**Abbreviations**: SATS, South African Triage Scale; RETTS, Rapid Emergency Triage and Treatment System; LMIC, Low- and Middle-Income Country; LMICs, Low- and Middle-Income Countries; ICC, Intraclass Correlation Coefficient; κ, Kappa Coefficient.

### Quantitative and Qualitative Results

The synthesis of quantitative and qualitative findings (Table 2) demonstrates that standardized nurse-led triage systems produce consistently positive effects across multiple dimensions of road-traffic and trauma care. The most reproducible gains were observed in triage accuracy, timeliness, and clinical outcomes. Across the eight included studies, nurses achieved sensitivity levels of 70–92% with under-triage rates ≤20%, while waiting times and on-scene-to-care intervals were reduced by up to 45% and 38%, respectively. Notably, mortality reduction and stabilization improvements were most evident in settings implementing validated frameworks such as the South African Triage Scale (SATS), Rapid Emergency Triage and Treatment System (RETTS), and digital prehospital triage applications.

A key contextual finding from Kenya highlighted that, despite high diagnostic accuracy, the RETTS validation study revealed persistent assessment delays in the absence of a nurse-led triage process, underscoring the essential role of trained nursing leadership in ensuring timely evaluation.^29^ Implementation feasibility was consistently demonstrated even under severe resource constraints, though barriers such as staff shortages, infrastructure limitations, and incomplete documentation remained recurrent. Furthermore, several studies reported marked improvements in nurse confidence, professional autonomy, and interprofessional coordination following formal institutionalization of triage leadership.

Collectively, these findings reinforce that structured nurse-led triage is both effective and scalable, improving diagnostic precision, care timeliness, and patient outcomes while simultaneously strengthening professional nursing roles within emergency and prehospital systems in low- and middle-income settings.

### Risk of Bias Assessment

**Figure 2:**
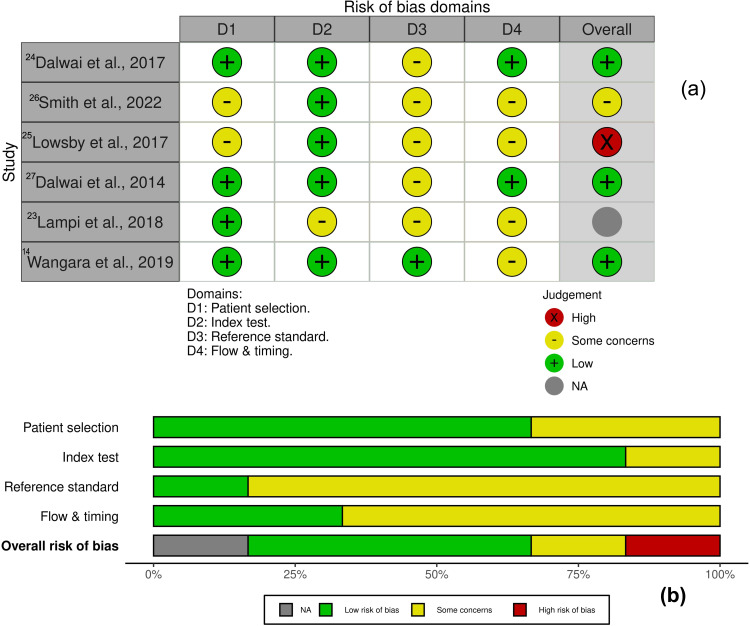
Risk-of-Bias Summary Across Study Designs. (**a**) QUADAS-2 traffic-light plot showing domain-level judgments for diagnostic-accuracy studies^17^,^25–29^ (**b**) QUADAS-2 summary bar plot displaying the percentage distribution of low risk (green), some concerns (yellow), high risk (red), and not-applicable (gray) ratings across the four domains.

Risk of bias was assessed using design-specific appraisal frameworks, with findings indicating generally acceptable methodological quality across the included studies. Under the QUADAS-2 tool, six diagnostic-accuracy studies^17^,^25–29^ demonstrated overall low-to-moderate risk of bias. Most domains, including patient selection and index-test conduct, were rated low risk owing to the use of standardized nurse-led triage protocols such as the South African Triage Scale (SATS) and Rapid Emergency Triage and Treatment System (RETTS). Dalwai et al (2014) and Wangara et al (2019) showed particularly low risk across index-test and reference-standard domains, confirming high diagnostic accuracy and reliability in trauma-dominant LMIC settings.^17^,^28^ Minor concerns were identified in the reference-standard and flow-and-timing domains, primarily where comparator data or follow-up intervals were incomplete.^27^ The study by Lampi et al (2018) was rated “unclear” for the reference-standard domain because triage priority was correlated with Injury Severity Scores rather than an independent diagnostic gold standard.^29^ Overall, the QUADAS-2 summary plots reflected predominantly low-risk ratings with isolated “some concerns” and one study demonstrating high overall risk due to flow-and-timing and patient-selection limitations (Figure 2).

The single implementation study (Harrison et al, 2012), appraised using the ROBINS-I framework, exhibited a moderate overall risk, mainly due to potential confounding inherent in its before-and-after design, though intervention classification and outcome measurement were of low risk.^18^

The cross-sectional study (Mathew et al, 2024), evaluated with the JBI checklist, showed clearly defined inclusion criteria, consistent measurement, and sound analytical methods, yielding a moderate overall risk largely from uncontrolled confounders such as injury severity and prehospital delay.^24^

### Quality Appraisal and Risk of Bias Summary

The methodological quality of the eight included studies was overall moderate to high. Appraisal using the Joanna Briggs Institute (JBI), QUADAS-2, and ROBINS-I tools showed that three studies (38%) demonstrated high methodological quality, four (50%) were rated as moderate, and one (12%) as acceptable Common methodological strengths included clearly defined inclusion criteria, standardized triage protocols, and valid outcome measures with consistency between intervention and comparison groups. The main limitations were incomplete adjustment for potential confounders, modest sample sizes in single-center studies, and limited blinding or follow-up reporting in observational designs. One study was classified as having a high overall risk of bias, while the remaining studies demonstrated low to moderate risk. Overall, the evidence base was methodologically sound and appropriate for narrative synthesis within the LMIC trauma and road-traffic triage context. A detailed summary of individual study appraisals is presented in Supplementary Table 2.

## Discussion

This systematic review synthesized evidence from eight empirical studies evaluating standardized nurse-led triage protocols in road-traffic and trauma-care settings across low- and middle-income countries (LMICs). Collectively, the evidence demonstrates that structured triage systems—such as the South African Triage Scale (SATS), Rapid Emergency Triage and Treatment System (RETTS), and Field Triage Decision Scheme (FTDS)—consistently improved patient prioritization, timeliness, and stabilization outcomes. Reported benefits included reductions in under-triage (≤20%), improved triage accuracy (κ = 0.72–0.88; ICC = 0.77), and shorter triage-to-decision intervals (20–45%).^17^,^25^,^27–29^ In Kenya, validation of RETTS demonstrated a strong correlation between assigned acuity levels and injury severity but also revealed delays in the absence of consistent nurse leadership, underscoring the importance of trained nursing oversight for timely evaluation.^29^ Similarly, SATS validation studies across LMICs confirmed strong inter-rater reliability and effective acuity differentiation by trained nurses, reinforcing the feasibility and diagnostic precision of standardized triage in trauma-dominant settings. In prehospital systems, nurse–paramedic collaboration improved destination accuracy and reduced time to definitive care.^24^ Overall, these findings affirm that nurse-led structured triage is both feasible and clinically effective when adapted to local resource constraints.

Considerable variation was observed across the included studies in terms of design, implementation context, triage tools applied, and outcome reporting.^17^,^25^,^27^,^29^ Some investigations employed prospective or quasi-experimental designs in tertiary emergency departments, whereas others conducted retrospective validation or service-evaluation studies in district hospitals or prehospital systems.^8^,^27^ While the South African Triage Scale (SATS) was most frequently evaluated,^17^,^25^ RETTS and other context-adapted frameworks were implemented in selected regions.^6^,^29^ Reported outcomes also varied, with some studies emphasizing diagnostic accuracy and inter-rater reliability,^26^ while others focused on waiting-time reduction, stabilization indicators, or mortality correlations.^8^,^11^ These contextual and methodological differences informed the decision to synthesize findings narratively rather than quantitatively.

Findings from this review align with previous literature recognizing the central role of nurses in strengthening LMIC emergency-care systems.^3^,^4^ Earlier studies largely identified systemic deficiencies in triage reliability and training, whereas the present synthesis quantifies measurable outcome gains linked specifically to nurse-led structured triage. Implementation of SATS across African contexts yielded reproducible improvements in mortality reduction and acuity classification reliability.^8^,^17^,^30^ Comparable benefits were reported with RETTS- and RTTDC-based frameworks, which enhanced recognition of people injured in road-traffic crashes and reduced waiting times.^31^,^32^ These outcomes are consistent with the World Health Organization (WHO) Emergency Care Systems Framework and the International Federation for Emergency Medicine (IFEM) Quality Standards,^33^ both of which identify structured triage as a cornerstone of safe, equitable, and efficient trauma care.

The effectiveness of nurse-led triage in LMICs is supported by three synergistic mechanisms: clinical standardization, task redistribution, and system integration. Standardized decision-making reduces subjective variability by utilizing objective physiological parameters—such as vital signs, consciousness level, and injury mechanism—to assign reproducible color-coded acuity levels.^26^,^34^ Task redistribution to trained nurses addresses physician shortages and enables rapid stabilization of people injured in road-traffic crashes, improving workflow and care delivery.^12^,^35^ Ongoing professional development through programs such as ETAT+ and SATS refresher courses sustains skill retention and adherence to triage algorithms.^36^ System integration, including structured documentation, supervision, and feedback loops, reinforces continuity and accountability.^37^,^38^

Evidence from emergency departments in LMICs shows that validated triage tools are most effective when paired with empowered and accountable nursing leadership.^6^,^9^ Across all reviewed contexts, standardized triage systems generated not only clinical gains but also system-level improvements. Facilities implementing SATS or RETTS demonstrated shorter waiting intervals, enhanced patient flow, and more efficient use of emergency resources.^13^,^17^ These structured systems strengthened interfacility coordination and reduced misclassification, supporting continuity of trauma care even within resource-limited environments. Additionally, formal recognition of triage as a nursing-led responsibility fostered greater professional autonomy, morale, and adherence to evidence-based protocols.^39^,^40^ These psychosocial benefits further enhance quality and sustainability within emergency-care systems.

Nonetheless, several implementation barriers persist. Limited access to structured training, high staff turnover, inadequate supervision, and resource shortages-particularly of monitoring equipment and documentation tools-were recurrent challenges.^20^,^41^ Weak communication linkages between prehospital and hospital services also impeded triage fidelity. However, institutions that embedded triage within continuous quality-improvement (QI) cycles demonstrated stronger sustainability, compliance, and adaptability^8^ These insights underscore that long-term success depends not only on clinical training but also on institutional support and policy-level commitment to nursing-led emergency-care models.

Key strengths of this review include rigorous adherence to PRISMA 2020 standards, comprehensive multi-database searching, and independent dual-reviewer screening, ensuring transparency and reproducibility. By focusing exclusively on low- and middle-income countries (LMICs) and resource-limited emergency-care environments, this review provides contextually relevant insights for global trauma-care policy and practice. However, heterogeneity in study designs, triage instruments (SATS, RETTS, FTDS, and digital systems), and outcome measures precluded meta-analysis and limited direct comparability. The predominance of observational, validation, and quasi-experimental designs limits the ability to establish definitive cause-and-effect relationships between nurse-led triage implementation and improved patient outcomes. Moreover, incomplete documentation of implementation fidelity and cost-effectiveness constrained full assessment of scalability. The review was restricted to English-language publications, which may have excluded relevant studies published in other languages and introduced potential language bias. Despite these limitations, convergence of evidence across diverse contexts and methodologies supports the robustness, validity, and generalizability of nurse-led structured triage as an effective approach for improving trauma-care performance in LMICs. Although formal application of the GRADE framework was considered, the substantial heterogeneity in study designs, outcome measures, and implementation contexts precluded structured grading of certainty across pooled outcomes. Instead, the overall strength of evidence was interpreted narratively, taking into account methodological quality, consistency of findings, and contextual applicability. Across the included studies, the direction of effect was consistently favorable toward standardized nurse-led triage, supporting moderate overall confidence in the conclusions.

Integrating nurse-led triage into national emergency-care frameworks can substantially strengthen trauma outcomes and system responsiveness in LMICs. Nursing curricula should include structured triage modules and simulation-based training to reinforce rapid decision-making and prioritization skills. Implementation strategies must prioritize scalability, sustainability, and cost-effectiveness, leveraging existing nursing workforce capacity and hierarchical supervision. Embedding triage indicators into national health information systems will enable continuous monitoring, benchmarking, and quality improvement, aligning with Sustainable Development Goal (SDG) 3.6-to halve global road-traffic deaths by 2030.

Based on the synthesized evidence, nurse-led triage should be institutionalized within national emergency and trauma-care frameworks as a central strategy for system strengthening. Structured triage and simulation-based learning should be integrated into both undergraduate and continuing nursing education to sustain competence. Health institutions must maintain supervision, documentation, and audit-feedback systems to ensure adherence and performance monitoring. At the system level, low-cost digital and mobile triage tools can enhance decision support and coordination between prehospital and hospital services. Governments and academic institutions should promote scalable, cost-effective models that leverage existing nursing capacity while enabling safe task-shifting. Multi-country research collaborations assessing long-term outcomes, cost-effectiveness, and scalability are recommended to guide evidence-based policy. Collectively, these measures can strengthen emergency-response capacity, reduce trauma-related mortality, and advance progress toward SDG 3.6.

Future research should assess the long-term effectiveness, sustainability, and economic impact of nurse-led triage using cluster-randomized or stepped-wedge designs. Rigorous controlled designs are particularly needed to clarify causal pathways and quantify the direct impact of nurse-led structured triage on mortality and morbidity outcomes in trauma populations. Studies evaluating digital and AI-assisted triage platforms, real-time feedback systems, and mobile decision-support tools are needed to enhance accuracy, integration, and scalability. Mixed-methods investigations exploring leadership engagement, institutional integration, and cultural adaptability will help identify determinants of sustained performance. Regional collaborations linking African, South Asian, and Pacific emergency networks could establish shared benchmarks and competency frameworks to build triage capacity and promote cross-country learning. These initiatives will be critical to achieving system-wide standardization and strengthening global trauma-care resilience.

## Conclusion

This systematic review provides compelling evidence that standardized nurse-led triage frameworks demonstrate consistent improvements the effectiveness, timeliness, and accuracy of trauma and road-traffic injury care in resource-limited and LMIC settings. Structured triage tools such as SATS, RETTS, FTDS, and RTTDC consistently reduce under-triage, improve patient prioritization, and shorten care intervals, underscoring the pivotal role of nurses as frontline decision-makers where physician coverage is limited. Beyond clinical outcomes, nurse-led triage strengthens system coordination, efficiency, and equity, aligning with WHO Emergency Care Systems and IFEM Quality Standards. Empowering nurses through standardized triage protocols represents a sustainable, evidence-based strategy for improving patient safety, quality, and system resilience in trauma care across low-resource environments.
